# A Microfluidic Device for Massively Parallel, Whole-lifespan Imaging of Single Fission Yeast Cells

**DOI:** 10.21769/BioProtoc.2783

**Published:** 2018-04-05

**Authors:** Stephen K Jones, Eric C Spivey, James R Rybarski, Ilya J Finkelstein

**Affiliations:** 1 Department of Molecular Biosciences and Institute for Cellular and Molecular Biology, The University of Texas at Austin, Austin, TX, USA; 2 Center for Systems and Synthetic Biology, The University of Texas at Austin, Austin, TX, USA; 3 Department of Biomedical Engineering, Vanderbilt University, Nashville, TN, USA

**Keywords:** Cellular aging, Lifespan, Microdissection, Microfluidics, Lithography, Fabrication

## Abstract

Whole-lifespan single-cell analysis has greatly increased our understanding of fundamental cellular processes such as cellular aging. To observe individual cells across their entire lifespan, all progeny must be removed from the growth medium, typically via manual microdissection. However, manual microdissection is laborious, low-throughput, and incompatible with fluorescence microscopy. Here, we describe assembly and operation of the multiplexed-Fission Yeast Lifespan Microdissector (multFYLM), a high-throughput microfluidic device for rapidly acquiring single-cell whole-lifespan imaging. multFYLM captures approximately one thousand rod-shaped fission yeast cells from up to six different genetic backgrounds or treatment regimens. The immobilized cells are fluorescently imaged for over a week, while the progeny cells are removed from the device. The resulting datasets yield high-resolution multi-channel images that record each cell’s replicative lifespan. We anticipate that the multFYLM will be broadly applicable for single-cell whole-lifespan studies in the fission yeast (*Schizosaccharomyces pombe*) and other symmetrically-dividing unicellular organisms.

## Background

Cellular aging results in the cumulative decline of cellular function that eventually leads to mortality. Most studies of cellular aging focus on the replicative lifespan of model unicellular organisms, such as budding yeast *Saccharomyces cerevisiae* (Nyström and Liu, 2014; Wasko and Kaeberlein, 2014; Wierman and Smith, 2014; Ruetenik and Barrientos, 2015). The replicative lifespan (RLS) of a cell is defined as the number of daughters produced by a mother cell over the course of its life (Henderson and Gottschling, 2008; Sutphin *et al*., 2014). RLS studies have greatly expanded our understanding of cellular aging in mitotically active cells. For example, in budding yeast, old mothers preferentially retain misfolded proteins and other cellular senescence factors from the budding daughter cells (Aguilaniu *et al*., 2003; Hughes and Gottschling, 2012; Liu *et al*., 2010; Saka *et al*., 2013; Zhou *et al*., 2014; Paoletti *et al*., 2016). This feat is achieved by restricting the flow of these ‘senescence factors’ across the bud septum, preventing their accumulation in the rejuvenated daughters (Shcheprova *et al*., 2008; Higuchi-Sanabria *et al*., 2014). Whether senescence factors are also segregated in symmetrically dividing cells is unclear (Wang *et al*., 2010; Coelho *et al*., 2013; Nakaoka and Wakamoto, 2017). Indeed, relatively little is known about the mechanisms and causes of aging in symmetrically dividing cells.

Whole-lifespan cellular aging studies require the separation of aging cells from their progeny. Pioneering, early studies in budding yeast removed daughter cells from their mothers via manual microdissection (Mortimer and Johnston, 1959). Since the first such study in 1959, manual microdissection still remains a popular, albeit laborious method for studying replicative aging in most unicellular organisms (Mortimer and Johnston, 1959; Kennedy *et al*., 1994; Barker and Walmsley, 1999; Fu *et al*., 2008). However, the low-throughput and laborious nature of this assay limits our current understanding of replicative aging. Most recently, removal of progeny cells has been automated in microfluidic devices that capture and retain individual aging cells (Wang *et al*., 2010; Lee *et al*., 2012; Xie *et al*., 2012; Zhang *et al*., 2012; Tian *et al*., 2013; Crane *et al*., 2014; Nobs and Maerkl, 2014; Jo *et al*., 2015; Liu *et al*., 2015; Nakaoka and Wakamoto, 2017; Spivey *et al*., 2017). Using such devices, relatively large cohorts of individual cells (100 s to 1,000 s of cells) can then be tracked independently from one another. However, most of these approaches focused on prokaryotic cells or the asymmetrically dividing budding yeast (Spivey and Finkelstein, 2014; Chen *et al*., 2017).

Here, we describe the fabrication and assembly of a microfluidic device for capturing and imaging thousands of fission yeast cells over their entire replicative lifespans. The multiplexed fission yeast lifespan microdissector (multFYLM) enables the experimentalist to track the lifespan of over a thousand fission yeast cells (Spivey *et al*., 2017). The cells may be continuously imaged for up to six independent populations for over a week, yielding high-resolution imaging over each cell’s replicative lifespan. The multFYLM is constructed of silicone elastomer using templates manufactured via UV photo-lithography. The protocol contained herein details construction of the multFYLM, loading with fission yeast cells, and image collection using a fluorescent microscope. We anticipate that this protocol will be broadly useful for long-term imaging of rod-shaped eukaryotic cells and will shed light on diverse biological processes, including cell cycle regulation, chromatin dynamics, proteome homeostasis, and cellular aging.

## Materials and Reagents

### A. Microfabrication

1. SU-8 2005 photoresist (Microchem)
2. SU-8 2010 photoresist (Microchem)
3. P-doped silicon wafers (University Wafers, catalog number: 452; 100 mm-diameter; test-grade)
4. Custom quartz photomasks (Compugraphics)
  Photomask design files available at: https://github.com/finkelsteinlab/FYLM_mask_files/raw/master/l1-151030.gds
  https://github.com/finkelsteinlab/FYLM_mask_files/raw/master/l2-151030.gds
5. SU-8 developer (Microchem)
6. Acetone (Pharmco-Aaper, Midland Scientific, catalog number: 329000000CSGF)
7. Isopropanol (Fisher Chemical, catalog number: BP26184)
8. Cyclopentanone (Sigma-Aldrich, catalog number: W391018-1KG-K)

### B. multFYLM assembly

1. 50 ml conical tubes (Genesee Scientific, Olympus Plastics, catalog number: 21-108)
2. Large Petri dish (150 mm; Fisher Scientific, catalog number: FB0875714)
3. 200 μl pipette tips (Genesee Scientific, catalog number: 23-150RL)
4. Biopsy punch (P125; 1 mm Acu-punch; Acuderm)
5. Glass coverslips (48 × 65 mm #1; Gold Seal, Thermo Fisher Scientific, Thermo Scientific™, catalog number: 3335)
6. Aluminum foil (Fisher Scientific, catalog number: 01-213-102)
7. Razor blades (duridium style, single edge; Gem/Star)
8. Lab tape (Fisher Scientific, catalog number: 15-901-10R)
9. Nanoports (IDEX Health & Science, catalog number: N-333; 12 x; 10–32 thread; headless knurled head)
10. Polydimethylsiloxane (PDMS; Dow Corning, Sylgard 184, Fisher Scientific, catalog number: 50-366-794)
  Manufacturer: Electron Microscopy Sciences, catalog number: 2423610.
11. Hellmanex III (Hellma Analytics)
12. Ethanol (Pharmco-Aaper, catalog number: 111000200CSPP)

### C. Microscope and microfluidics setup

1. PFA Tubing (IDEX Health & Science, catalog number: 1512L; 1/16″ OD)
2. Coned nut and ferrule (IDEX Health & Science, catalog number: F-333N; 12 x; 10–32 thread; headless knurled head)
3. Inline filter (IDEX Health & Science, catalog number: P-272; 6 x)
4. Luer adapter (IDEX Health & Science, catalog number: P-658; 6 x; ¼–28 thread; Luer-Lok thread)
5. Flangeless nut (IDEX Health & Science, catalog number: P-215; 6 x; ¼–28 thread)
6. Union (IDEX Health & Science, catalog number: P-704-01; 6 x; 10–32 thread)

### D. Cell loading and image acquisition

1. Test tubes (14 ml; Corning, catalog number: 352051)
2. Large gauge syringe needles (16 G 1.5”; BD, catalog number: 305198)
3. Large syringes (100 ml; Veterinary Concepts, catalog number: 60271)
4. 10 ml syringes (Luer-Lok tip; BD, catalog number: 309604)
5. Steriflip vacuum filtration tubes (50 ml; 20 μM nylon net; Millipore Sigma, catalog number: SCNY00020)
6. Petri dishes (100 mm; Fisher Scientific, catalog number: FB0875713)
7. Stericup-GP filter sterilizing module (500 ml; 0.22 μm PES; Millipore Sigma, catalog number: SCGPU05RE)
8. Yeast strains
9. Bovine serum albumin (Sigma-Aldrich, catalog number: A2153)
10. Agar powder (Sigma-Aldrich, catalog number: A1296)
11. YES 225 powder (250 g; Sunrise Science, catalog number: 2011-250)
12. YES 225 agar media (Recipe 1)
13. YES 225 liquid media (Recipe 2)

## Equipment

### A. Microfabrication

1. 1 L flask (No. 1000; Corning, Pyrex^®^, catalog number: 1000-1L)
2. Suss MA-6 Mask Aligner (Suss MicroTec Lithography GmbH)
3. Spin Coater (Laurell Technologies)
4. Hot plate (Cimarec+; Thermo Fisher Scientific, Thermo Scientific, catalog number: HP88857100)
5. Anisotropic RIE Plasma Etcher (Nordson March, catalog number: CS170IF)
6. Hot-Hand Protector Mitt (SP Scienceware - Bel-Art Products - H-B Instrument, catalog number: F38000-0001)

### B. multFYLM assembly

1. Mini labroller (Labnet)
2. Plasma Cleaner (Harrick Plasma, catalog number: PDC-32G)
3. Laboratory oven (Ecotherm, Precision)
4. Dissection microscope (AmScope, catalog number: SM-1T-PL)
5. Fine tweezers (Fisher Scientific, catalog number: 16-100-103)
6. Sonicator (Bransonic, catalog number: 2510R-DTH)
7. Rocker/agitator (Belly Dancer; Stovall Life Science)
8. Bunsen burner (accuFlame; Fisher Scientific, catalog number: 03-902Q)
9. Centrifuge (Beckman Coulter, model: Avanti^®^ J-26XP)
10. Centrifuge rotor (Beckman Coulter, model: JLA-16.250)

### C. Microscope and microfluidics setup

1. Epifluorescence imaging microscope (Eclipse Ti; Nikon)
2. Focus maintenance system (Nikon Perfect Focus, Nikon)
3. CMOS camera (Andor, model: Zyla 5.5 sCMOS)
4. 10x, 0.3 NA objective (Plan Fluor; Nikon)
5. 60x, 0.95 NA objective (Plan Apo Lambda; Nikon)
6. Computer-controlled microscope stage (Proscan III motorized stage; Prior)
7. Objective heater (Bioptechs, catalog number: 150819-19)
8. Appropriate filters for fluorescent imaging
  - eGFP (Chroma, catalog number: 49002)
  - mKO (Chroma, catalog number: 49010)
  - E2Crimson (Chroma, catalog number: 49015)
9. Light source shutter (SmartShutter; Sutter Instrument)
10. Shutter controller (Lambda SC; Sutter Instrument)
11. Computer-controlled syringe pump (KD Scientific, model: LEGATO^®^ 210)
  Note: This pump is configured for two syringes. If more than two syringes are required, either multiple pumps can be used, or adapters can be fabricated (Figure 4) to allow additional syringes to be driven.
12. Light source (Newport, model: SOLA-SE-II; Lumencorp)

### D. Cell loading and image acquisition

1. Shaking incubator (Thermo Fisher Scientific, Thermo Scientific™, catalog number: 4333)
2. Spectrophotometer (Thermo Fisher Scientific, Thermo Scientific™, model: NanoDrop™ 2000c)
3. Autoclave (Consolidated Sterilizer Systems, model: ADV-PLUS)
4. Vacuum desiccator (5.8 L Pyrex glass; Corning, PYREX^®^, catalog number: 3121-200)
5. Vacuum pump (Welch Vacuum, catalog number: 2546, B-01)
6. Mini vortexer (Fisher Scientific, catalog number: 02-215-365)
  Note: This product has been discontinued.
7. Bunsen burner (accuFlame; Fisher Scientific, catalog number: 03-902Q)
8. Environmental chamber/multFYLM microscope stage
  Chamber design file available at: https://github.com/finkelsteinlab/FYLM_mask_files/blob/master/FYLMChamber.scad

## Software

1. NIS-Elements Advanced Research (v4.30.02; Nikon Instruments)

## Procedure

### A. Microfabrication

**Figure 1:**
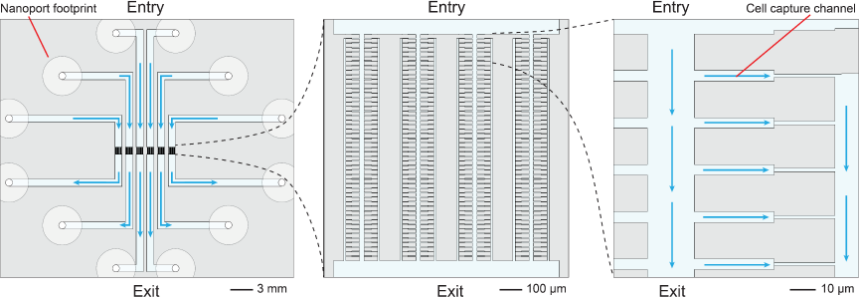
Overview of the multFYLM design The multFYLM contains six independent paths. Media enters through each nanoport at the top of the device (Entry), and then follows the path indicated by blue arrows, before exiting through nanoports at the bottom of the device (Exit).

multFYLM microfabrication follows conventional soft lithography methods. The first step is to generate a patterned mold, which can be used to cast devices in elastomeric silicone (PDMS). Such molds, or ‘master’ structures are created on silicon wafers, using UV lithography to deposit patterns on the surface in an epoxy resin (SU-8). The patterns are dictated by masks, which restrict the ability of a UV light source to cross-link the resin. Their alignment is critical to the proper patterning on the wafer, as features of the final master are contained on each of the two masks. A developer is used to remove unexposed resin, leaving a master that is now ready for use (Figure 1). A master can be used repeatedly for at least two years to make hundreds of multFYLM devices.

Note: The procedures detailed below should be performed in a cleanroom. All instrument settings are unique to the equipment used and included as a guideline. These settings will need to be adjusted to match the instruments available in a user’s cleanroom. All microfabrication steps should be completed in a single day; although suitable stopping points may exist, they have not been tested.

1 Rinse the polished wafer surface with acetone, isopropanol, and then water.
2 Air-dry the wafer while setting up the plasma cleaner.
3 Set the hotplate to 200 °C.

#### Plasma cleaning

Plasma clean the wafer to yield an ultra-clean surface, so that resin patterns may be deposited on the surface with high resolution and adherence.

4 Turn on the plasma cleaner and gas controller.
5 Create a plasma cleaning program (Table 1) that will clean the wafer with a 30/70 ratio of O_2_ to N_2_. More time does not necessarily yield a better surface.
6 Break the chamber vacuum, and load the wafer with the polished side up.
  Note: Manual operation works best for bleeding the vacuum.
7 Run the cleaning program.
  - Change the RF tuning switch to Auto, then start the program.
  - Reverse power flow should be minimized during plasma flow, via adjustment of the C1 and C2 switches.
  - Upon program completion, allow vacuum bleeding to finish, then stop the program.
8 Remove wafer.
9 Re-establish chamber vacuum to promote instrument longevity and cleanliness.
10 Turn off components.

#### Prepare the mask aligner

Turn on the mask aligner components, so they can equilibrate before use.

11 Turn on gas and vacuum lines.
12 Turn on the mask aligner.
13 Start UV lamp–it requires a 10-min warm up period.

#### Prepare wafer for first exposure

Deposit the first layer of resin evenly on the wafer surface to yield a resin thickness of 5–6 μm. Alignment, spin parameters and resin application are all critical for proper deposition.

14 Place the wafer directly on a hotplate with the polished surface face up for 20 min at 200 °C.
  Note: This step assures that the wafer is dry. The temperature of the wafer does not have to be maintained once removed from the hotplate, but one should proceed quickly to the next step. A hot-hand protector mitt may be used to transfer the wafer between instruments.
15 Place a 100-mm carousel on the spin coater.
16 Carefully transfer the wafer to the very center of the carousel, opening the vacuum line to firmly hold the wafer in place. An off-center wafer will not yield an even layer of SU-8 in Step A21.
17 Turn on the spin coater, and set the spin coater program:
  - 10 sec at 500 rpm, acceleration level 2 (266 rpm/sec).
  - 35 sec at 1,500 rpm, acceleration level 4 (532 rpm/sec).
18 Run the program, adding two drops of cyclopentanone to the wafer surface once the speed reaches 1,500 rpm.
19 Add 6 ml of SU-8 2005 resin to the wafer surface as evenly as possible–avoid dripping SU-8 over the sides of the wafer.
20 Wait 3 min while bubbles rise to the surface of the SU-8 on the wafer.
21 Run the program from Step A17.
22 The cover should be lifted slowly to avoid dripping SU-8 onto the freshly-spun wafer.
23 Dampen a wipe with cyclopentanone and remove the SU-8 bead remaining on the edge of the wafer surface. Alternatively, an edge-bead removal protocol may be used if the spin coater is so equipped.
24 Release vacuum pressure and remove the wafer from the carousel.
  Note: The resulting layer of SU-8 should be uniform. If not, the wafer must be cleaned with isopropanol and the procedure restarted from Step A1.
25 Heat the wafer from room temperature to 95 °C on a hotplate that is initially off.
26 Leave the wafer on the hotplate at 95 °C for 4 min.
27 Turn off the hotplate and let the wafer cool down on it for 10 min.

#### Expose wafer with the first mask

Install the first mask and the resin-covered wafer into the mask aligner. Expose the wafer to UV light long enough to produce patterns in the resin at sufficient resolution. Under-exposure results in incomplete patterning or diminished features, while over-exposure results in enlarged features and low resolution.

28 Adjust the mask aligner parameters (Table 2). The parameters here should only be used as a guideline.
29 Set mask 1 into the mask holder.
  - Remove and install the correct mask holder for 100-mm wafers.
  - Set the mask in the holder chrome-side face up, using vacuum to hold the mask in.
30 Position the mask in the approximate center of the viewable region.
31 Load wafer into the wafer holder.
32 Align wafer with the mask, using alignment marks as a guide.
33 Expose the wafer to UV light, and wait for the exposure to complete.
34 Remove the wafer.

#### Remove unexposed photoresist from the wafer

Use developer to remove the unexposed resin from the wafer surface; this process reveals the deposited features. Excessive developing will cause the deposited features to be washed off.

35 Place wafer back on a cooled hotplate.
36 Heat up to 95 °C, then incubate at that temperature for one minute.
37 Place wafer in a 1 L flask photoresist-side up.
38 Pour developer over wafer to cover it completely.
39 Allow developing to proceed for 30 sec with agitation.
40 Remove wafer.
41 Rinse wafer surface with fresh developer.
42 Rinse wafer surface with isopropanol.
43 Dry the wafer using pressurized N_2_.

#### Prepare the wafer for second exposure

Deposit the second layer of resin evenly on the wafer surface to yield a resin thickness of 20–30 μm. Both the resin and spin parameters have been optimized for depositing a resin layer with the proper characteristics for the second exposure.

44 Clean the wafer surface in the plasma cleaner following Steps A4–A10 but with the following program (Table 3).
45 Place the wafer directly on a hotplate with the polished surface face up for 20 min at 200 °C.
  Note: This step assures that the wafer is dry. The temperature of the wafer does not have to be maintained once removed from the hotplate, but one should proceed quickly to the next step. A hot-hand protector mitt may be used to transfer the wafer between instruments.
46 Place a 100-mm carousel on the spin coater.
47 Carefully transfer the wafer to the very center of the carousel, opening the vacuum line to firmly hold the wafer in place. An off-center wafer will not yield an even layer of SU-8 in Step A21.
48 Set the spin coater program:
  - 14 sec at 500 rpm, acceleration level 2 (266 rpm/sec).
  - 37 sec at 3000 rpm, acceleration level 4 (532 rpm/sec).
49 Add 6 ml of SU-8 2010 to the wafer surface as evenly as possible–avoid dripping SU-8 over the sides of the wafer.
50 Wait 9 min while bubbles rise to the surface of the SU-8 on the wafer.
51 Close cover and run the program from Step A48.
52 The cover should be lifted slowly to avoid dripping SU-8 onto the freshly-spun wafer.
53 Dampen a wipe with cyclopentanone and remove the SU-8 bead remaining on the edge of the wafer surface. Alternatively, an edge-bead removal protocol may be used if the spin coater is so equipped.
54 Release vacuum pressure and remove the wafer from the carousel.
55 Heat the wafer from room temperature to 85 °C on a hotplate that is initially off.
56 Leave the wafer on the hotplate at 85 °C for 15 min.
57 Turn off the hotplate and let the wafer cool down on it for 10 min.

#### Expose wafer with the second mask

Install the second mask and the resin-covered wafer into the mask aligner. Expose the wafer to UV light long enough to produce patterns in the resin at sufficient resolution. Alignment at this step is critical, as it ensures that features produced using the second mask will overlay properly with those already on the wafer surface.

58 Adjust the mask aligner parameters (Table 4). The parameters here should only be used as a guideline.
59 Set mask 2 into the mask holder.
  - Remove and install the correct mask holder for 100-mm wafers.
  - Set the mask in the holder chrome-side face up, using vacuum to hold the mask in.
60 Position the mask in the approximate center of the viewable region.
61 Load wafer into the wafer holder.
62 Adjust the position of the wafer such that it is aligned with the mask, using the alignment marks on the second mask and the wafer (from the first exposure).
63 Expose the wafer to UV light, and wait for the exposure to complete.
64 Remove the wafer.

#### Remove unexposed photoresist from the wafer

Use developer to remove the unexposed resin from the wafer surface. This process reveals the deposited features. Excessive developing will cause the deposited features to be washed off.

65 Place wafer back on a cooled hotplate.
66 Heat up to 85 °C, then incubate at that temperature for ten minutes.
67 During the incubation, remove the second mask from the mask aligner, and then turn off the mask aligner and UV lamp.
68 Place wafer in a 1 L flask photoresist-side up.
69 Pour developer over wafer to cover it completely.
70 Allow developing to proceed for 3 min with agitation.
71 Remove wafer.
72 Rinse wafer surface with fresh developer.
73 Rinse wafer surface with isopropanol.
74 Repeat Steps A72–A73.
75 Dry the wafer using pressurized N_2_.

### B. multFYLM assembly

Assembly of the multFYLM via soft-lithography proceeds once the master structure is complete. The master structure is used as a mold for PDMS. Before the PDMS hardens, ports are added to allow media flow into the microfluidic structures. Once the silicone has set, it is cleaned and adhered to a large cover glass. The thin, transparent cover glass forms the base of the multFYLM and allows imaging of cells that are captured within the individual arms of the device.

#### Cast the multFYLM in polydimethylsiloxane (PDMS)

**Figure 2:**
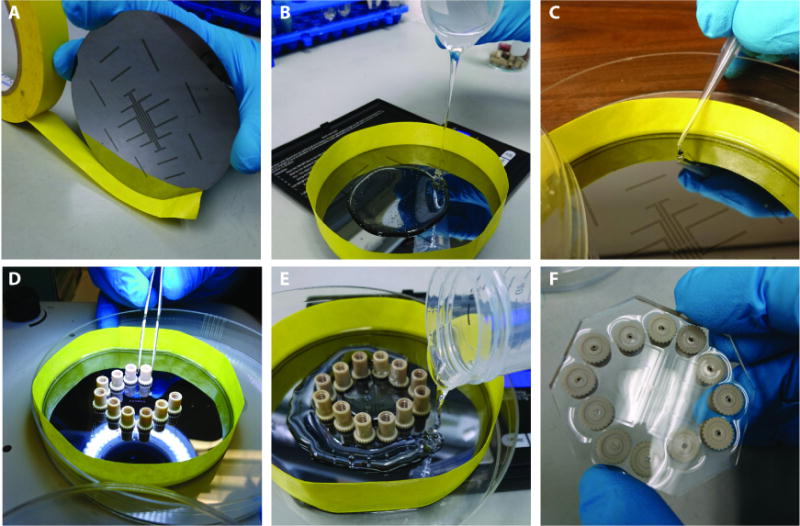
Soft lithography A. Paper tape surrounds the wafer containing the SU-8 master to keep the PDMS in place while it sets. B. First layer of PDMS. C. Layer one is semi-hardened. D. Nanoports are placed on the first layer. E. The second PDMS layer is poured around the nanoports. F. The fully-cured multFYLM, removed from the master structure.

Prepare a PDMS solution according to the manufacturer’s protocol (Figure 2). Wrap the master structure with tape to create a vertical barrier for the PDMS. Pour half of the solution onto the master structure. Soft-bake the first layer until it is tacky, then place a clean port over each conduit passage present on the master structure. Pour the remaining PDMS onto the first layer, then bake it until all the PDMS has fully hardened.

1 Preheat oven to 75 °C.
2 Mix 30 g of PDMS with 3 g of the hardening agent in a 50 ml conical tube. Install the cap.
3 Mix the PDMS solution on a lab roller for 45 min at room temperature.
4 Clean 12 nanoports in 2% Hellmanex in a bath sonicator for 20 min on the sonication setting (nanoports can also be cleaned ahead of time).
5 Rinse the nanoports thoroughly in filtered DI water.
6 Place the nanoports in 70% EtOH in the bath sonicator for 20 min on the sonication setting.
7 Create a barrier around the circumference of the patterned wafer, using standard lab tape.
  - At least 2 mm of the tape should extend evenly below the circumference of the wafer.
  - Tape adhesion is critical in order to avoid PDMS leaking from the wafer surface.
8 Set the wafer inside a large (150 mm) Petri dish.
9 Centrifuge the mixed PDMS solution at room temperature at > 400 × *g* for 90 sec to remove large bubbles.
10 Pour 13 g PDMS onto the wafer, allowing it to wet the entire surface evenly.
11 Dry the nanoports at 70 °C on a hotplate for 30 min.
12 Remove residual air bubbles by placing the wafer in a vacuum desiccator for 15 min at 60–70 cmHg.
13 Remove the vacuum rapidly to remove bubbles still trapped in the PDMS. Repeat if necessary.
14 Place the wafer with PDMS in the oven at 70 °C for 15 min.
15 Test the PDMS on the wafer for proper hardness.
  - PDMS should be semi-solid and very tacky, making a small peak when probed with a 200 μl pipette tip.
  - If not, place it back in the oven, checking every few minutes for proper hardness.
16 Using a dissection scope, delicately place each of the twelve nanoports over the end of each media conduit of the PDMS as seen in the wafer’s pattern.
  Avoid placing a nanoport down more than once on the PDMS surface. Multiple placements can damage multiple fluid passages, and a misaligned port may prevent fluid flow to the corresponding passage.
17 Pour 14 g PDMS onto the wafer, allowing it to wet the entire surface evenly.
18 Place the wafer in a vacuum desiccator for 15 min at 60–70 cmHg. This removes any air that may be trapped under the nanoports, ensuring a good seal between the nanoports and the PDMS.
19 Return the wafer to the oven (70 °C) for 3 h, or until fully cured.

#### Cut, clean and assemble the multFYLM

Remove the multFYLM from the master structure, then use a razor blade to trim away excess PDMS. Use a biopsy punch to make a direct path from each molded conduit to the nanoport on the opposite side of the multFYLM. Ultraclean the multFYLM and a large cover glass, then adhere them to one another. This completes assembly of the multFYLM.

20 Place a cover glass in a Petri dish containing a 2% Helmannex solution for one hour with agitation on a rocker.
21 Rinse the cover glass twice with diH_2_O.
22 Rinse the cover glass twice with isopropanol.
23 Place the cover glass in a large Petri dish containing a single layer of aluminum foil.
24 Set the dish on a hotplate at 70 °C for at least two hours to dry.
25 Carefully remove the tape from the circumference of the patterned wafer and multFYLM.
26 Carefully peel the cast multFYLM from the wafer surface.
  - Peel gently as to avoid splitting the polymerized PDMS.
  - Do not touch the surface that was in contact with the patterned wafer.
27 Invert the multFYLM in the Petri dish, ports-side down.
28 Cut away excess PDMS from the patterned/ported region of the multFYLM using a new, sharp razor blade.
29 Position the multFYLM under a dissection microscope.
30 Using a 1 mm biopsy punch, gently punch a hole through the center of each nanoport from the bottom surface through to the top surface.
  Note: The punched region should include the conduit that the nanoport was placed over.
31 Remove the ‘punched-out’ core of PDMS from the biopsy punch using fine tweezers before withdrawing the biopsy punch from the multFYLM.
32 Remove the biopsy punch from the multFYLM with light pressure and a slight rotating motion to avoid separating the nanoport from the PDMS.
33 Inspect the nanoports and remove any remaining PDMS particles with the fine tweezers.
34 Submerge the multFYLM in a beaker containing 100% isopropanol and place the beaker in the bath sonicator for 30 min on the sonication setting.
35 Remove the multFYLM from the beaker and place it ports-side down in a large Petri dish containing a single layer of aluminum foil.
36 Set the dish on a hotplate at 70 °C for two hours to dry.
37 Place the recently-dried multFYLM and cover glass in the plasma cleaner, with the surfaces that will contact the cover glass facing up.
38 Turn on the plasma cleaner.
39 Turn on the vacuum to evacuate the chamber for at least one minute.
40 Turn the RF setting to ‘high’ for 20 sec.
41 Immediately remove the components from the plasma cleaner.
42 Adhere the cover glass and multFYLM by carefully setting the cleanest side of the cover glass onto the center of multFYLM.
43 Apply light pressure to the multFYLM to assure that it has fully-adhered to the cover glass.
  - For best results, the multFYLM should be used within several hours of assembly. Alternatively, the multFYLM may be stored in 70% ethanol for extended, sterile storage.
  - The completed multFYLM should be stored in a container to avoid contamination.

#### Microscope and microfluidics setup

Whole-lifespan imaging adds additional technical challenges to operating any microfluidic device. First, the microfluidic system must provide fresh media to the captured cells while also removing waste. Imperfections in the flow path can cause air bubbles that dislodge cells, potentially disrupting a multi-day experiment. Moreover, additional precautions must be taken to remove cells that are trapped upstream of the multFYLM. This is because these cells may grow into microcolonies during multFYLM operation, ultimately obstructing the flow of fresh media to the device. Second, the microscope should be equipped with stable optical and mechanical components for up to a week of continuous imaging. An active feedback focus-finding system ensures that the multFYLM can be imaged for several days without requiring any user intervention. Similarly, a light source (*i.e*., LED lamp) that does not change in output intensity or spectrum during a week of continuous operation is recommended. Finally, we recommend that the entire device is enclosed in an incubator jacket that maintains optimal growth conditions for the desired cells (see Equipment D8).

#### Prepare microfluidic tubing

**Figure 3:**
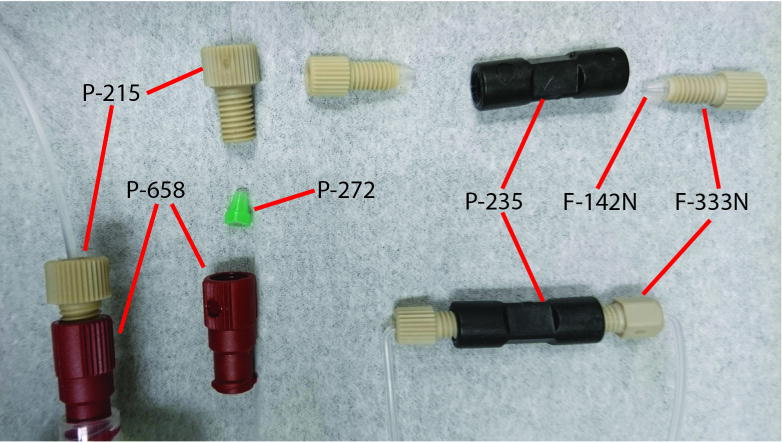
Microfluidic fittings

Clean all the microfluidic fittings (Figure 3) that will be used for attaching to the multFYLM, then fit them onto microfluidic tubing. It is necessary to put a right angle in the tubing immediately after the fittings that will attach to the nanoports, otherwise the tubing will not clear the environmental chamber and microscope components.

44 Submerge all microfluidic fittings in a beaker containing 2% Hellmannex detergent and sonicate in a bath sonicator for 20 min on the sonication setting.
45 Rinse all fittings with diH_2_O three times.
46 Submerge all microfluidic fittings in a beaker containing 100% ethanol and sonicate in a bath sonicator for 20 min on the sonication setting.
47 Rinse all fittings with 100% ethanol.
48 Dry all the fittings in a Petri dish on a hotplate at 70 °C for 30 min or longer.
49 Cut twelve sections of tube to the length of 60 cm. Cut ends to be as square as possible.
50 Using a Bunsen burner as an aid, permanently bend a 95° angle into one end of each tube, approximately 17 mm from the end.
51 For six tubes that will become the waste lines, attach the following fittings at the bent end:
  - F-333N coned nut, threads away from the bend.
  - F-142N ferrule, blunt end towards the bend. The tubing should extend beyond the ferrule by 1–2 mm.
52 For six tubes that will become the media lines, attach the following fittings:
  - F-333N coned nut, threads away from the bend.
  - F-142N ferrule, blunt end towards the bend. The tubing should extend beyond the ferrule by 1–2 mm.
  - P-215 flangeless nut, threads toward the straight end.
  - P-272 ferrule, blunt end away from the flangeless nut.
  - P-658 Luer adapter, screwed onto the flangeless nut, sandwiching the ferrule.
53 Connect each media line to a waste line using P-235 connectors.
  Note: Tubing should be prepared ahead of time, and can be stored in ethanol or sterile water until use.

#### Prepare the microscope for imaging

Turn on the microscope and peripherals, so that they can warm up before the experiment begins. The NIS Elements software (or other control software) should also be opened, as some peripherals may not turn on completely without a signal from the correctly-configured software.

54 Turn on the following components:
  - Microscope
  - Camera
  - Shutter controller
  - Stage
  - Objective heater–set to achieve 30 °C within the multFYLM. The heater should be installed on the 60x air objective. The temperature setting should be determined empirically, as a higher programmed temperature will likely be required to account for heat loss.
  - Stage heater–set to achieve 30 °C within the multFYLM. The temperature setting should be determined empirically, as a higher programmed temperature will likely be required to account for heat loss.
  - LED light source
  - White light source
55 Start the NIS Elements software suite.
  - Select ‘Neo/Zyla’ as the image grabber if prompted.
  - Move the 10x objective into position.

### C. Cell loading and image acquisition

Below, we describe a protocol to maximize the number of cells that are captured in the multFYLM. Since the multFYLM contains many fine passages, it can become clogged with cell clumps or other debris. Care must be taken while preparing and loading the media and cells to avoid any particles or cell clumps. Further, air can easily dislodge captured cells, and so it should be purged from any upstream components in the fluid path. Use sterile techniques to prevent other microbes from contaminating cells in the multFYLM.

Image acquisition of cells in the multFYLM requires image collection at dozens of locations, regular time intervals, multiple Z planes, and filters corresponding to the range of fluorophores present. While an in-focus Z plane is used for the majority of imaging, the out-focus Z plane allows for greater certainty in defining the cell boundaries. Care should be taken when selecting fluorophores and filters, as spectral separation allows for unambiguous attribution of fluorescence to individual fluorophores.

#### Prepare media and cells

Make a liter of degassed, filtered YES 225 media, and culture the yeast strains so that they will be in exponential growth-phase on the first day of the experiment.

1 Prepare one liter of YES 225 agar media (Recipe 1).
2 Prepare one liter of YES 225 liquid media (Recipe 2).
3 Prepare 1 ml of sterile 20% BSA solution in a conical tube.
4 Streak cells from glycerol stocks onto the agar plates four days prior to the start of the experiment. Plates should be incubated at 30 °C until colonies are well-formed, then left at room temperature.
5 Select a 2–3 day-old colony and inoculate 10 ml of YES 225 media in a test tube.
6 Incubate the cell culture overnight in a shaking incubator at 30 °C.
7 When the optical density at 595 nm (OD_595_) of the cell culture reaches 0.1, inoculate a fresh test tube containing 10 ml of YES 225 media.
8 Incubate the new cell culture in a shaking incubator at 30 °C until the OD_595_ is 0.5 to 1.0 (4–6 h).
9 Degas the YES 225 media by placing it in a vacuum desiccator with the bottle cap loose for 15 min. This should be done just prior to loading the media into syringes.

#### Connect and clean media/waste lines

**Figure 4:**
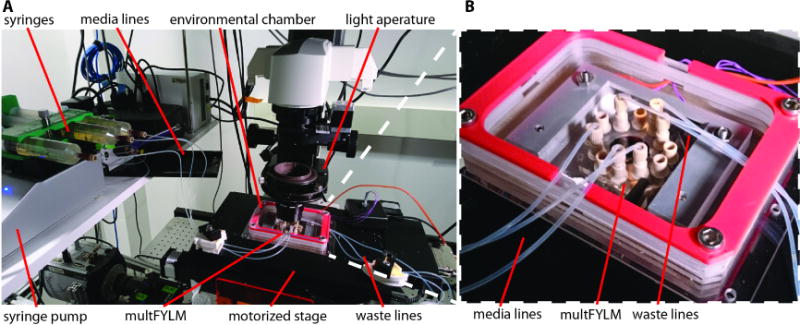
Epifluorescent microscope prepared for imaging of the multFYLM A. The complete multFYLM microfluidic path. B. Microfluidic fittings connect lines to the multFYLM.

Load the prepared media into syringes large enough to hold enough media for the entire experimental time course. Clean the media and waste lines using ethanol and sterile water, as sterility is essential to experimental success. Install the multFYLM in the environmental chamber, then connect the waste lines (Figure 4).

10 Turn on the syringe pump.
11 Determine how many flowpaths within the multFYLM will be used.
  Only three or four of the available six flowpaths are typically used due to spatial constraints and image collection rates. All six flowpaths can be used if the image collection rate is infrequent enough, the lines do not over-torque the multFYLM, and all areas can be observed by the microscope.
12 Fill N 10 ml syringes (‘N’ equal to as many flowpaths that will be used) with 70% ethanol.
13 Load these syringes into the syringe holder on the syringe pump.
14 Connect N media/waste line sets to each ethanol syringe.
15 Set the syringe pump parameters and run:
  - Syringe: B-D Plastipak 10 ml syringe
  - 5 min
  - 1 ml/min
16 Fill N 10 ml syringes with diH_2_O.
17 Replace the ethanol syringes with the water syringes.
18 Rerun the pump according to Step C15.
19 Load N syringes with the degassed YES media.
  - Attach a large syringe needle to the syringe to aid in loading the syringe without introducing any air bubbles.
  - Any air in the syringes should be removed immediately.
20 Replace the water syringes with the YES media syringes.
21 Set the syringe pump parameters and run it to replace the water in the lines with YES media:
  - Custom syringe–diameter 31.75 mm
  - 1 min
  - 1 ml/min
22 Retrieve the multFYLM and attach it to the heated stage insert using spring metal clips or lab tape.
  By convention, the multFYLM is oriented as parallel to the imaging area as possible, with entrance ports oriented closest to the user. The entrance ports lead to the end of the microfluidic pattern that is not directly accessible to the waste trenches at the periphery of the channels intended to hold the cells.
23 Detach the waste lines from media lines and attach them to the exit channels of the multFYLM.
  - Take care to avoid placing lines over regions that will be imaged during the experiment.
  - Connecting lines to all six paths concurrently is difficult. It is generally advisable to run no more than three or four flowpaths in parallel.
  - Be sure to perform this task in as sterile a manner as possible.
  - Media lines should be kept sterile until connected. Storing them in an open conical tube is typically sufficient to prevent contamination.

#### Load cells into the multFYLM

Carefully vortex and load cells into each entry port, then attach the media lines while avoiding introduction of any air. Establish a program for the syringe pump that typically provides a consistent flow rate, with an occasional, increased flow rate; this will help dislodge any debris that might otherwise clog the passages of the multFYLM.

**Figure 5:**
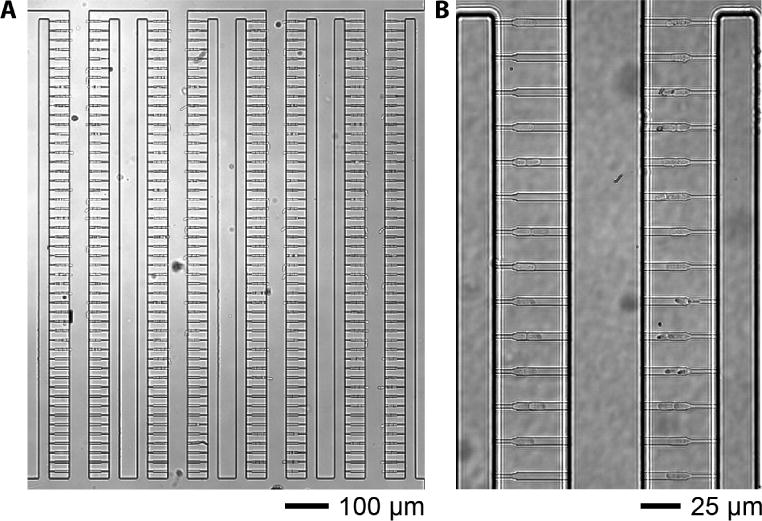
*Schizosaccharomyces pombe* cells loaded into the multFYLM A. 10× image of cells within a single flowpath immediately following the loading process. B. 60x image of cells viewable within the defined region of interest (ROI).

24 Transfer 400 μl of each cell culture into separate microfuge tubes.
25 Add 100 μl of sterile 20% BSA solution to each tube.
26 Vortex each tube for one minute.
27 Using a micropipette, transfer 40 μl of cell solution to each appropriate entry port.
  - Take care to introduce as little air as possible. This volume assures that enough liquid is present to allow a drop-to-drop connection with the media line without over-filling the nanoport during setup.
  - The pipette tip should be held just above the base of the port to avoid introducing air to the flowpath.
28 The cells may be observed using white light and the 10x objective. They should begin to flow into the multFYLM due to surface tension.
29 Set the syringe pump at a rate of 40 μl/min and run.
30 As YES media begins to exit each media line, gently attach it to an entry port.
  Be very careful while attaching: use a drop-to-drop connection strategy to avoid introducing air to the flowpaths, and do not torque on the multFYLM. Ports can easily separate, or the cover glass can crack.
31 Observe the cells using white light and the 10x objective. They should be filling the channels of the microfluidic flowpath, starting near the entry ports first (Figure 5A).
32 Create a program for the pump with the following parameters:
  - One minute at a flow rate of 55 μl/min
  - Fourteen minutes at 5 μl/min.
  - Repeat 725 times.
33 Once cells have filled most of the channels to be observed, start the above program.

#### Begin image acquisition

Using the NIS Elements software, set up a multi-dimensional acquisition strategy that will capture images of cells in each compartment of the multFYLM at regular time intervals, an in-focus and out-of-focus Z plane, and all filter sets necessary for the emission of the fluorophores in use (Table 5). Other software suites may be used, though the following directions are specific to NIS Elements.

34 Move the 60x air objective into place.
35 Obtain focus, then turn on the Perfect Focus System (PFS) using the PFS button on the front of the microscope.
36 Using the stage controller, bring the left-most flowpath in use into view.
37 Change the Region of Interest (ROI) to the same size as the viewable area of cell channels.
  - In the Zyla settings menu in NIS Elements, use the ‘Commands’ > ‘ROI’ > ‘Load ROI’ drop-down menu and then select the *.CAMROI file downloaded from below.
    Camera ROI file: https://github.com/finkelsteinlab/FYLM_mask_files/blob/master/FYLM_ROI_2X2_new.camroi
  - In the same sub-menu, select ‘Use current ROI’ (Figure 5B).
38 Under the ND Acquisition menu, set the folder and file names.
  - Path: Location where the generated files will be stored.
  - Filename: Name of the files to be generated. A three-digit number will be appended to the end automatically.
39 Under the ND Acquisition menu, set Time:
  - The interval and total duration of the image collection. Frequency is dependent on the number of channels that will be collected, but for white light-only images, a 2 min interval is reasonable.
  - It is recommended that the duration be 36 h or less, to balance file size with image collection restart frequency.
40 Under the ND Acquisition menu, set Z:
  - In-focus (as determined using PFS)
  - 4 μM offset (Step: 4, 2 steps, Below: −4, Above: 0)
41 Under the ND Acquisition menu, set λ:
  - Optical configurations should be set up for each fluorescent image filter set. Exposure times should be determined experimentally.
  - Select all optical configurations that will be used during the experiment.
  - Fluorescent images do not need to be collected at every time period (reduces the likelihood of photo-toxicity), and frequency can be set using T Pos.
  - Z-depth is selectable. It is recommended that fluorescent images only be collected at the ‘Home’ Z Pos.
42 Under the ND Acquisition menu, set XY:
  - X-Y positions should be tiled across the observable cells.
  - It is recommended that positions be defined in a loop pattern to avoid large changes in the focal plane, which can lead to loss of focus mid-experiment.
43 Once all parameters have been set (Table 5), press ‘Run’.
44 Observe the first few rounds of imaging to assure that everything remains as set.
45 The experiment should be observed at least once a day to check for errors and to collect a new image file. Downstream analysis is optimized for files containing 24 h of data.
46 After 24 h, press ‘Finish’ to complete one day’s collection.
  - This will also save the file, though saving can be assured by accessing ’Save’ in the ‘File’ menu.
  - If prompted, it is not necessary to complete the current loop before finishing.
47 Image analysis software may now be used to create videos and analyze the collected data.

## Data analysis

Information on how data collected using this methodology is analyzed can be found in these references (Greenstein *et al*., 2017; Spivey *et al*., 2017).

## Notes

1. During microfabrication, the type and volume of photoresist, and the spin parameters can be varied to alter the height of the deposited features. Similarly, the type of photoresist and exposure time and intensity can be varied to alter the resolution and width of the deposited features. This can be particularly useful for capturing cells with slightly larger dimensions.
2. A common failure point during multFYLM assembly is punching out the PDMS from the center of the nanoports. Often, removal of the punch results in the nanoport lifting away from the PDMS, creating a small pocket of air. With care, such pockets of air can later be expelled when the coverglass is pressed to the PDMS. If pockets remain, they can become a reservoir for air that will dislodge cells while passing through the multFYLM, or for other cells that can clump and block the passageways as the experiment proceeds.
3. Loading the multFYLM with cells often works best with a freshly-assembled device, as the interior is still quite dehydrated, thus media and cells readily flow into it in order to rehydrate the surfaces. If the multFYLM has been stored for a length of time, it is advisable to run one ml of 70% ethanol, then one ml of water through the device backwards, so that air is not trapped in the exit channels. Otherwise, trapped air will not be displaced from the exit pathways, and adjacent channels will not yield the required pressure differential necessary for subsequent cell loading.

## Recipes

1. YES 225 agar media (1 L)
  - 36.13 g YES 225 powder, 20 g agar; add diH_2_O up to 1 L total volume
  - Autoclave, then pour 25 ml into individual Petri plates using sterile technique
2. YES 225 liquid media (1 L)
  - 36.13 g of the YES 225 powder; add diH_2_O up to 1 L total volume.
  - Filter sterilize the solution–this will also remove small particulates that can lead to clogged passages. Autoclave treatment is not sufficient, as it will sterilize the solution but will not remove particulates

## Figures and Tables

**Table 1:** First plasma cleaning program

Pressure	0
Power	150 W
EndPt	100 sec
Temp	25 °C
BP/RP	90
R4(O_2_)	30%
RF tuning switch	Manual

**Table 2:** Mask aligner parameters for the first layer

Exposure	95 mJ/cm^2^
Mode	Vacuum
PreVac	15 sec
FullVac	10 sec
Purge time	10 sec
WEC	Continuous

**Table 3:** Second plasma cleaning program

Pressure	0
Power	100 W
EndPt	20 sec
Temp	25 °C
BP/RP	90
R4(O_2_)	30%

**Table 4:** Mask aligner parameters for the second layer

Exposure	150 mJ/cm^2^
Mode	Vacuum
PreVac	15 sec
FullVac	10 sec
Purge time	10 sec
WEC	Continuous

**Table 5:** Example parameters for multi-dimensional image acquisition

Dimensions		T’(140) × XY(16) × Z(2) × λ(4)	
Camera Name		Andor Zyla VSC-01632	
		1.2	
Numerical Aperture		1.333	
Number of Picture Planes		4	
**Plane #1**		**Plane #2**	
Name	Brightfield	Name	GFP
Component Count	1	Component Count	1
Modality	Widefield Fluorescence	Modality	Widefield Fluorescence
Camera Type	Andor Zyla	Camera Type	Andor Zyla
Binning	2 × 2	Binning	2 × 2
Exposure	100 msec	Exposure	150 msec
Readout Mode	Global shutter at 16-bit	Readout Mode	Global shutter at 16-bit
Readout Rate	200 MHz	Readout Rate	200 MHz
Conversion Gain	Dual Gain 1/3	Conversion Gain	Dual Gain 1/3
Spurious Noise Filter	on	Spurious Noise Filter	on
Sensor Mode	Normal	Sensor Mode	Normal
Trigger Mode	Internal	Trigger Mode	Internal
Temperature	−0.4 °C	Temperature	−0.4 °C
Microscope	Ti Microscope	Microscope	Ti Microscope
	2 (49002 - ET-GFP (FITC/Cy2))		2 (49002 - ET-GFP (FITC/Cy2))
	1	Shutter, Shutter(EPI-BF)	Closed
	Active	Shutter1, Shutter(EPI-FL)	Active
PFS-S, state	Off	PFS-S, state	Off
PFS-S, offset	6729	PFS-S, offset	6729
PFS-S, mirror	Inserted	PFS-S, mirror	Inserted
Zoom	1.00x		
**Plane #3**		**Plane #4**	
Name	mKO	Name	E2Crimson
Component Count	1	Component Count	1
Modality	Widefield Fluorescence	Modality	Widefield Fluorescence
Camera Type	Andor Zyla	Camera Type	Andor Zyla
Binning	2 × 2	Binning	2 × 2
Exposure	150 msec	Exposure	150 msec
Readout Mode	Global shutter at 16-bit	Readout Mode	Global shutter at 16-bit
Readout Rate	200 MHz	Readout Rate	200 MHz
Conversion Gain	Dual Gain 1/3	Conversion Gain	Dual Gain 1/3
Spurious Noise Filter	on	Spurious Noise Filter	on
Sensor Mode	Normal	Sensor Mode	Normal
Trigger Mode	Internal	Trigger Mode	Internal
Temperature	−0.4 °C	Temperature	−0.4 °C
Microscope	Ti Microscope	Microscope	Ti Microscope
	1 (49014 - ET-mKO/mOrange)		4 (49015 - Alexa 633)
PFS-S, state	Off	PFS-S, state	Off
PFS-S, offset	6729	PFS-S, offset	6729
PFS-S, mirror	Inserted	PFS-S, mirror	Inserted
		Time Loop	140-Nonequidistant
		Z Stack Loop	2 - Step 4 μm
